# Academic Health Science Centres as Vehicles for Knowledge Mobilisation in Australia? A Qualitative Study

**DOI:** 10.34172/ijhpm.2020.247

**Published:** 2020-12-12

**Authors:** Alexandra Edelman, Robyn Clay-Williams, Michael Fischer, Roman Kislov, Alison Kitson, Ian McLoughlin, Helen Skouteris, Gillian Harvey

**Affiliations:** ^1^College of Public Health, Medical and Veterinary Sciences, James Cook University, Townsville, QLD, Australia.; ^2^Australian Institute of Health Innovation, Macquarie University, Sydney, NSW, Australia.; ^3^Centre for Sustainable Human Resource Management and Wellbeing, Australian Catholic University, Melbourne, VIC, Australia.; ^4^Manchester Metropolitan University, Manchester, UK.; ^5^The University of Manchester, Manchester, UK.; ^6^Caring Futures Institute, College of Nursing and Health Sciences, Flinders University, Adelaide, SA, Australia.; ^7^Monash Business School, Monash University, Melbourne, VIC, Australia.; ^8^Monash Centre for Health Research and Implementation, Monash University, Melbourne, VIC, Australia.; ^9^Warwick Business School, University of Warwick, Coventry, UK.; ^10^Adelaide Nursing School, University of Adelaide, Adelaide, SA, Australia.; ^11^Australian Centre for Health Services Innovation, Queensland University of Technology, Brisbane, Australia.

**Keywords:** Academic Health Centre, Knowledge Mobilisation, Research Translation, Research Impact, Australia

## Abstract

**Background**: Despite increasing investments in academic health science centres (AHSCs) in Australia and an expectation that they will serve as vehicles for knowledge translation and exchange, there is limited empirical evidence on whether and how they deliver impact. The aim of this study was to examine and compare the early development of four Australian AHSCs to explore how they are enacting their impact-focused role.

**Methods**: A descriptive qualitative methodology was employed across four AHSCs located in diverse health system settings in urban and regional locations across Australia. Data were collected via semi-structured interviews with 15 academic, industry and executive board members of participating AHSCs. The analysis combined inductive and deductive elements, with inductive categories mapped to deductive themes corresponding to the study aims.

**Results**: AHSCs in Australia are in an emergent state of development and are following different pathways. Whilst varied approaches to support research translation are apparent, there is a dominant focus on structure and governance, as opposed to action-oriented roles and processes to deliver strategic goals. Balancing collaboration and competition between partners presents a challenge, as does identifying appropriate ways to evaluate impact.

**Conclusion**: The early stage of development of AHSCs in Australia presents an important opportunity for formative learning and evaluation to optimise their enactment of knowledge mobilisation processes for impact.

## Background

**Key Messages**

** Implications for policy makers**

- Academic health science centres (AHSCs) are academic-industry collaborations, with a specific aim to close translational gaps from discovery research to application in health policy and practice. Australian AHSCs are in a formative stage of development.
- The current focus within Australian AHSCs on high-level structuring around broad translational goals risks neglecting important “on the ground” systems and processes for effective knowledge mobilisation.
- AHSCs should move quickly to adopt organisational learning processes to avoid path dependency and optimise their impact potential.

** Implications for the public**

Academic health science centres (AHSCs) in Australia aim to improve health service delivery and patient and population health by better linking scientific research with healthcare and policy. However, little is known about whether and how this is achieved. The findings of this research show that Australian AHSCs are not yet fully embracing important tools used elsewhere to bring research closer to patients. The Australian public will benefit from efforts by AHSCs to learn from their own experiences to date and what has worked well in other countries.

The intentional development of academic health science centres (AHSCs) represents a move to bring universities and healthcare providers together in the pursuit of excellence in clinical service, research and education, with a particular focus on collaborating across traditional silos to promote innovation and research translation.^1^ Influenced by developments internationally, the case for AHSCs in Australia emphasised a need to improve translational links between basic science and clinical medicine.^2^ Formal establishment of AHSCs in Australia occurred through the creation of National Health and Medical Research Centre (NHMRC)-designated “Advanced Health Research and Translation Centres” (AHRTCs) and “Centres for Innovation in Regional Health” (CIRHs). Four AHRTCs were designated by the NHMRC in 2015, followed by a further three in 2017, alongside three CIRHs (designated in 2017 and 2019) that have a specific focus on regional Australian populations. The term “regional” in relation to CIRHs refers to locations outside of major metropolitan areas that may include regional cities as well as rural, remote and very remote townships and communities.

 Despite the growth of AHSCs, the majority of published literature is normative and originates from the United States, in the form of general commentaries, opinion pieces and case studies of individual AHSCs.^5^ Further, while there is a broad expectation that AHSCs will serve as vehicles for knowledge translation and exchange,^5^ empirical evidence of their impact is limited, as are more detailed insights into what works, how and why in terms of their specific translational processes and outcomes.^3,4^ Specific impact expectations vary widely: for example, while local, clinical impacts are a dominant focus, a substantial body of expert opinion also highlights the potential for AHSCs to contribute to “global health” by bringing healthcare and academic institutions together across countries to develop joint strategies to address healthcare challenges.^6^

 Whilst there is no “one size fits all” formula to AHSC success,^7^ an external evaluation of the Collaborations for Leadership in Applied Health Research and Care, established in England from 2008 onwards, highlighted eight important considerations to optimise the success of these types of collaborative initiatives.^8^ Key issues highlighted included the governance framework, leadership approaches, attention to evaluation and learning and balancing the tension between collaboration and competition (Table 1). The concept of “knowledge mobilisation” frames these considerations and is central to the mission of AHSCs as it reflects a two-way process of knowledge development and exchange between the partners involved.^9^

**Table 1:** Achieving Collective Action for Implementation: A Mid-Range Theory^4^

**Theme**	**Description**
Working relationships	If these are well-developed, or if there is pre-formative investment to develop them, this is likely to lead to quicker wins, increased appreciation of others’ positions, creating a platform upon which to build plans and activities.
Attention to evaluation and learning	If attention is lacking and/or leadership teams are not reflective, the initial interpretation of the mission can create a path dependency that is difficult to alter. Therefore, it is important to build in mechanisms for evaluation, learning and meta-learning to enable adaptation to changing contexts.
Governance framework	If this facilitates opportunities for physical, social and intellectual connectivity between stakeholders, it enables productive conversations and conducive conditions for implementation-related activities that resonate with partners.
Vision and strategy	A shared vision that is aligned across stakeholders in relation to knowledge production and use can unblock barriers to purposeful collective action.
Motivation for engagement	If the ‘what’s in it for me’ motives are made visible, implementation activity can be planned so that engagement is appropriately incentivised.
Boundary spanning	If resources are invested in boundary spanning mechanisms, such as credible knowledge broker and facilitator roles and the development of boundary objects, this can help to bridge boundaries and catalyse implementation activity.
Collaboration versus competition	Tension between collaboration and competition can act as both a facilitative or inhibitory force. As such, it is important to find the right balance between the two.
Leadership	There is a need for both strong central and distributed leadership as this facilitates collaboration and the potential for implementation.

 Compared to the United States, United Kingdom and Canada, AHSCs in Australia are at an earlier stage of development. This presents two distinct opportunities: firstly, it allows Australian AHSCs to draw on prior international learning to influence and shape their ongoing development and growth; secondly, it permits formative learning from highly diverse urban/regional contexts that characterise Australian AHSCs and adds to the international knowledge base about AHSCs.

 The overall aim of this study was to examine Australian AHSCs at their current stage of development in order to address the research question: how are people, processes and systems being organised within Australian AHSCs to enable knowledge to be mobilised for impact? The specific aims were to examine:

1. the strategic objectives of the AHSC in relation to achieving and demonstrating impact;
2. how board members think about the systems and processes required for effective knowledge mobilisation;
3. challenges encountered in mobilising knowledge to achieve impact; and
4. the potential for future research to inform strategies for enhancing knowledge mobilisation and impact in the AHSC.

## Methods

###  Study Design and Data Collection

 The research adopts a qualitative descriptive^10^ study design to describe the key features of interest and offer thoughtful linkages with other work in the field. Data were collected via semi-structured interviews with senior members and leaders of four AHSCs selected with reference to geographic and structural attributes as well as NHMRC designation status to enable consideration of different contextual features. Practical considerations including the researchers’ access to AHSC leaders and their capacity to support the study were also factors in the selection process. The participating AHSCs were comprised of two NHMRC-designated AHSCs and two that were non-designated. One AHSC was structured as a fully unified university-hospital, governed by a single Chief Executive and Board, while the other three AHSCs were multi-organisation collaborations. Two were regional AHSCs, encompassing large rural and remote geographies with highly distributed populations, while the other two were urban-based in large metropolitan cities.

 Interviews of between 30-40 minutes in duration were conducted by three members of the project team (GH, RCW, AE) with 15 academic, industry and executive board members in the participating AHSCs (Table 2). These individuals were purposively selected because they held strategic roles in developing the AHSCs and were able to reflect on both the origins and unfolding development of their AHSC. Researchers initially contacted these individuals by email or phone to request an interview, with all individuals initially contacted agreeing to be interviewed. The interview guide was developed with reference to the specific study aims and included questions about: strategic objectives; knowledge mobilisation structures and processes; challenges and barriers; and the perceived value of future impact-focussed research. Questions were deliberately broad to enable interviewees to shape the narrative about goals and activities of their AHSCs and built from the authors’ experiential knowledge of the field. Interviews were conducted in person or by video or telephone during 2019, and with the participants’ consent, were digitally recorded and transcribed verbatim. Transcripts were emailed to interviewees for checking.

**Table 2:** Study Sample

	**AHSC 1**	**AHSC 2**	**AHSC 3**	**AHSC 4**
Type of AHSC	NHMRC-designated	NHMRC-designated	Applying for NHMRC designation	Fully integrated university-hospital structure; outside NHMRC designation process
No. of interviewees	4	4	3	4
Representation	1 AHSC employee; 2 university representatives; 1 industry representative	1 AHSC employee; 3 university representatives	2 board members; 1 clinical academic	2 board members; 1 executive director; 1 clinical academic

Abbreviations: AHSC, academic health science centre; NHMRC, National Health and Medical Research Centre.

###  Data Analysis

 Data were entered into NVivo QSR^TM^ and initial inductive coding of the data from each of the four participating AHSCs was undertaken by two researchers (GH and AE) following reading and re-reading of the site-specific transcripts. An initial set of inductive codes for all four sites was then developed collaboratively through comparison of the researchers’ approaches and emerging findings. These codes were subsequently circulated to the broader team and inductive categories were then developed though virtual meetings and a full day face to face workshop. The workshop involved discussion among the researchers about emerging concepts and linkages following repeated reading of the transcripts.

 Consistent with the descriptive qualitative approach which seeks to provide an account of the experiences, events and processes of the phenomenon of interest,^10^ inductive categories reflected descriptive accounts of the AHSC goals, strategic processes and perceived enablers and barriers from the viewpoint of participants. Ultimately, the researchers determined that the best way to present the “emic” knowledge, or insider view,^10^ of interviewees, and to facilitate reporting of meaningful feedback of key study findings to participants, was to report the results against deductive themes produced from the question guide. These themes reflect the specific aims of the study and were informed by the literature on AHSCs and the authors’ experiential knowledge of the field.

 Because several members of the research team had some prior involvement with the AHSCs studied including as researchers and/or administrators within partnering organisations, the research team was already familiar with contextual elements such as funding and reporting structures and relevant national policy developments, which helped in the analysis process. These prior experiences also meant that some of the researchers were already known to the interviewees professionally and had already established rapport. To facilitate the inductive analysis, all members of the research team were closely involved in the careful reading and interpretation of the transcripts. Several members of the team also had experience conducting studies on AHSCs overseas which facilitated international comparison. Further, the analysis process drew from the diverse expertise of the research team in management and organisational studies as well as applied health services research to link findings with relevant theory and concepts across a range of disciplines. All members of the team had experience in designing and undertaking qualitative research.

## Results

 In the description of results below, interviewees are labelled using a random number within each AHSC, with AHSC identifiers reflecting their predominantly “urban” or “regional” location and orientation. Inductive categories are described under deductive themes reflecting the original specific aims of the study.

###  Strategic Objectives

####  Structures and Missions

 Objectives of the AHSCs broadly reflected the characteristic tripartite mission to undertake high quality research, education and care, and were also shaped by the AHSCs’ governance structures and location. While three of the AHSCs had built collaborative multi-organisational governance structures, one AHSC was modelled on the US approach, with a unified university-hospital structure. In the three multi-organisational AHSCs, there was a focus on working together at scale to deliver more than just *“the sum of the parts”* (Int 3, urban AHSC 1). In contrast, the goals of the integrated AHSC were influenced by its integrated nature and centred on creating a *“patient- centred culture”* and academic identity within a private hospital setting (Int 2, urban AHSC 2). Differences by urban/regional location were also apparent: interviewees in the regional AHSCs tended to highlight opportunities to improve health and outcomes for communities and populations, while the urban AHSCs were more focussed on patients in clinical settings.

####  Impact Through Research

 Across all sites, the establishment of the AHSC itself was a strategy to give greater structure and direction to the research endeavour of the component organisation/s, by shifting from researcher-led models to research co-produced with local stakeholders using the AHSC structure:

*“I think that’s the next stage, is to start to have the partners who are contributing, start to drive the strategy. What are the burning [issues] for them? Rather than have it as a bottom up, almost research-type strategy of investigator-led – [ ie,] what people want to do, rather than what perhaps the system needs. […] The Centre is helping to reinvigorate that with priority-driven-type research initiatives which I think is a very good thing” *(Int 1, urban AHSC 1).

*“Sometimes academics can be quite, I think, arrogant in their knowledge base, not deliberately but it’s very intimidating, kind of thing. So I think, you know, being mindful of who’s speaking and who isn’t speaking, and I think we’re moving forward on that [so that] service organisations are more confident in speaking out and speaking up” *(Int 3, regional AHSC 1).

 However, views on whether this was being achieved varied, even within the same AHSC. For example, one interviewee commented that *the “big-name researchers still call a lot of the shots”* in their AHSC and reflected that the centre felt like a series of discrete investigator-led projects (Int 1, regional AHSC 1); while others in the same AHSC believed that a history of working successfully together had enabled the centre to move quickly to priority-driven research informed by community needs (Int 2, regional AHSC 1).

 A wide and diverse range of research impact goals were described. For instance, some interviewees described a focus in their AHSC on practically focused research and translation for people and patients in clinical settings; while others described a concurrent focus on broader research goals linked to jurisdictional or national objectives, including commercialisation:

*“ There’s many objectives for various levels of strategic thinking […from a national perspective] the objectives are to create a network of quality health systems across the country that can lead the way in building translational research, [to] do clinical trials, and bring an export income, and create patents” *(Int 2, regional AHSC 2).

*“ So we are thinking about commercialisation, we are thinking about clinical trials, we are thinking about other things in the space, all sorts of things that we could be impacting on” *(Int 4, urban AHSC 1).

 One AHSC had international impact ambitions involving information sharing and collaboration with health system entities in South East Asia and the West Pacific in areas such as biosecurity, infectious diseases and health systems strengthening; the term *“intellectual leadership”* was used by an interviewee in this AHSC to describe this objective (Int 2, regional AHSC 2).

###  Systems And Processes For Knowledge Mobilisation

####  Enacting “Translation”

 Despite the wide range of objectives and impact goals described, the language of knowledge mobilisation was not generally used by interviewees. Most used the narrower concept of translation, referring predominantly to researcher-produced knowledge and its application in clinical contexts. Varied strategies to achieve translation were described, some of which were aspirational. These included demonstration or “flagship” projects, growing clinician research capacity and working through clinical leaders:

*“We’ve also got a selection of projects, some flagship projects, some projects that are MRFF [Medical Research Future Fund]-funded, and so there’ll be some case study stories to tell at a project level, and then there’ll be some, hopefully, case study stories we can tell at a centre level about how we’ve built the collaborations and moved people along a continuum” *(Int 4, urban AHSC 1).

*“Supporting people to do anything from a certificate through to a PhD to become an Early Career Fellow is what our aspirations should be, and we should be enabling some of that through the centre in big ways” *(Int 3, regional AHSC 1).

 Flagship projects were seen to be of value in the multi-organisational AHSCs as they encouraged collaboration to access project funding, while also providing a tangible outcome to showcase and learn from. Building clinician capacity to engage in research (through providing training, access to grants and clinical academic appointments) was also a dominant focus across all AHSCs. Building this capacity was described as a strategy to develop future clinician leaders (Int 2, urban AHSC 2), and came with the added benefit of enhancing recruitment capability within the participating health services:

*“You can attract really good doctors where there is research. Really good doctors like to do research as well as treat patients” *(Int 3, regional AHSC 2).

 Building research capacity and literacy among local clinicians and community was also described in one AHSC as a strategy to make research more responsive to community priorities:

*“The projects that have been rolled out in the sort of next round are very much around building capacity and literacy around research as part of the process rather than, say, looking at a particular condition or disease or, you know, health problem […] otherwise we’re just repeating the same thing again and again of, okay, a bunch of people in white coats think something about diabetes is interesting, so we’ll go and investigate it” *(Int 2, regional AHSC 1).

####  Governance (Re)structuring

 Across all the AHSCs, considerable attention had been directed to establishing high-level governance structures and, in some cases, revisiting and revising these. Governance in the multi-organisational AHSCs was typically concerned with ensuring adequate representation of participating partners to form a basis for effective collaboration, although this could lead to unwieldy decision-making processes. As a result, some AHSCs had opted for a functional structure, such as an executive or management committee and a (wider) council. In one AHSC, the ongoing evolution of the governance structure was seen to be indicative of its adaptive capabilities:

*“I’m happier with it as it evolves, so the fact that it has evolved has been a credit to all the people [involved] – that they take the initial model and […] they tried to make it more functional as it goes along. They recognise what it means to get to the next step” (Int 2, urban AHSC 1)*.

###  Challenges Encountered

####  Research Careers and Leadership

 Key developmental challenges encountered in the AHSCs included barriers to clinicians developing research careers within public facilities and the need for a shift in focus/culture within the fully integrated AHSC from clinicians’ private practice to quality improvement and research. Across all the AHSCs, recruiting to leadership roles – particularly roles that were intended to deliver value for the AHSC as a whole – was also seen as a challenge. Not only did such individuals need to be supported by all partners, the task of establishing and running an AHSC was described as complex. The need for a distinctive skill set was identified, directed towards relationship-building and flexibility, reflecting the complex nature of translational projects (in contrast to more time-limited project management skills):

*“You actually need to be really careful when you recruit staff. Because some people are not very good with a blank page, or a page that isn’t perfect, and they want a linear job. Those people don’t succeed in this environment” *(Int 4, urban AHSC 1).

####  Collaboration and Competition

 Another challenge, specific to the multi-organisational AHSCs, related to balancing collaboration and competition between the partnering organisations, including handling politics, diverse interests and egos. Whereas some sites reported a long history of working relationships that had created trust and a solid foundation for collaboration, others were working to overcome a history of competition. Bringing universities together with health service partners represented one challenging aspect. There was also a dynamic tension between collaboration and competition among academic organisations that had come together to apply for NHMRC designation, against a history of competing for research funding. One interviewee reflected that without the AHSC and the promise of NHMRC designation some of the emergent partnerships and collaborations would not have developed – it brought together several different groups who had previously competed:

*“I’d probably say it’s one of the difficulties out in rural, remote and very remote areas is that there’s a competitiveness to the research being conducted out there. So there wasn’t anything there before [the AHSC] but there were these distinct groups” *(Int 1, regional AHSC 1).

 Building the relationships needed to achieve impact goals was seen to require more than a well-developed governance framework. Interviewees in one AHSC emphasised the importance of shared values, expectations and trust; including a willingness to be *“prepared to transfuse your own blood”* (for example, in terms of brand, activity or people) to benefit the greater good:

*“In this sector, unless you’re going to give some of yourself, you’re not going to get stronger. I’m not sure whether the partners would be comfortable with the notion that actually they’re going to give some of themselves, some of their brand, some of their activity, some of their people, for the sum of the parts” *(Int 3, urban AHSC 1).

####  Government Funding

 There was widespread acknowledgement that successfully establishing the AHSC and achieving impact was challenging and would take time. However, this was contrasted with the short-term government funding received by the NHMRC-designated AHSCs, which necessitated a “rushed” process of allocation within the AHSCs (Int 4, regional AHSC 1).

###  Future Research to Maximise Impact Potential

####  Measuring Impact

 There was general interest across the AHSCs in research to both demonstrate impact and enable benchmarking between AHSCs; but how to measure this, including what metrics would be appropriate (or inappropriate), was another identified challenge. One interviewee described a temptation within AHSCs to measure narrow, mostly quantitative, impact indicators, which often appealed to health service administrators who tended to sit in executive roles and on AHSC boards:

*“You’re facing people whose bread and butter hasn’t been health system stuff, but [who] have done quite a bit of corporate stuff. And they want to see the numbers” *(Int 2, regional AHSC 2).

####  Interest in Evaluation Processes

 Overall, participants’ stated interest in engaging with evaluative forms of research varied, with responses ranging from enthusiasm for opportunities to share learning with other AHSCs, to uncertainty about the priority of evaluative research at a relatively early developmental stage.Interviewees in one AHSC commented that they were not good at “learning from other systems” (Int 1, urban AHSC 1), but that there should be some interest in formal evaluation and sharing learning across AHSCs, particularly the designated AHSCs which had received public funding.

## Discussion

 The findings of this study illustrate that AHSCs in Australia are in relatively early and formative stages of development, with individual AHSCs following different pathways. They operate in highly diverse contexts with varying degrees of scale and reach, which influences their strategic focus and impact goals. Differences in impact focus were identified between the urban and regional AHSCs, and between the multi-organisational AHSCs and the integrated AHSC. Diversity in what constitutes “success” is a recognised feature of AHSCs in Australia^3^ and indicates that a wide range of knowledge mobilisation processes are needed to achieve their varied academic, clinical, policy and population impact aspirations.

 However, understanding and enactment of knowledge mobilisation processes in the AHSCs studied were somewhat limited, with little attention being given to the complex and multifaceted realities shaping clinical and broader impact. Whilst the AHSCs studied described using several strategies to effect research translation in clinical settings, including research capacity building, participatory approaches, flagship projects and clinical engagement, there was little overall mention of processes of negotiation to systematise the utilisation of knowledge. Processes to integrate research production and use through negotiation between organisations and academic disciplines emerges as a key distinction between “knowledge translation” and “knowledge mobilisation” in the international literature.^9^ This distinction highlights the limitations of linear conceptions of knowledge flows between researchers and end users (which often underlie the “translation” concept) and instead emphasises the need for constant awareness and negotiation of complex inter-relationships.^9^

 Further, although there was a focus within the AHSCs on research capacity building within clinical settings and mention of goals to co-produce research, there was no specific reference within the four AHSCs studied to establishing knowledge broker or boundary spanner roles, which feature as key knowledge mobilisation processes elsewhere.^11,12^ Limited attention to these roles is particularly surprising given the emphasis identified in the AHSCs on structure and governance, as designated brokers and boundary spanners often play a critical linkage function between collaborating academic and healthcare organisations.^12^ These findings suggest important opportunities within Australian AHSCs to facilitate real-world impacts from research by trialling and adapting knowledge mobilisation processes tested in other settings.

 In the multi-organisational AHSCs, balancing collaboration and competition between constituent members presents a major challenge, mirroring international experiences.^5^ In the international literature, there is an emphasis on achieving a balance between two aspects of collaboration, defined as cooperation and coordination.^13^ Cooperation, concerned with achieving partners’ commitment and alignment of interests, tends to be highlighted, whereas less attention is given to the critical role of coordination activities directed to the alignment of actions to achieve shared goals. Successful coordination requires practices, structures, roles, procedures and interfaces that “prevent ad hoc responses to emerging problems.”^13^ For example, broad goals and commitments on their own are unlikely to be sufficient to organise interactions across organisational boundaries.^13^ As such, an over-emphasis on high-level agreements and goals in the pursuit of cooperation risks neglecting key systems and processes required for “on the ground” actions to mobilise knowledge.^9^

 Organisational learning that informs and shapes formative development is an important component of organisational capability in knowledge-intensive AHSCs.^14^ Given the scope for innovation and learning from Australian AHSCs’ richly diverse, regional and urban contexts, there is an important opportunity for AHSCs to learn and formatively steer towards success, rather than follow “path dependent” trajectories that can take a long time to correct.^8^ By creating, and acting on, a clear strategic vision as well as cohesion among partners, AHSCs in Australia can have real health system impacts.^3^ To optimise such impacts, Australian AHSCs should foster and embed a culture of formative evaluation that enables learning and adaptation, rather than rely narrowly on metrics-based evaluations. Future research on AHSCs should also investigate key mechanisms of knowledge mobilisation in diverse local contexts. Such research is needed to understand the role of AHSCs in responding to health system needs and priorities, such as in prepareness and response to major public health events such as the coronavirus disease 2019 (COVID-19) pandemic.

###  Strengths and Limitations

 Key strengths of the study include its novel focus on AHSCs as emerging health system structures in Australia and the use of international knowledge mobilisation literature to situate the study and interpret the findings. The study represents one of the first attempts to describe how AHSCs are developing in Australia across diverse health system contexts and offers a valuable insider perspective into the motivations and experiences of those involved in their strategic development. Limitations include the small number of interviewees from each AHSC, and their identification from within only one stakeholder group (individuals holding strategic roles in developing the AHSCs), which limited the depth of insights gained at each site. Nonetheless, the interviews provided rich and detailed findings appropriate to study aim. Inclusion of additional perspectives from researchers, clinicians, community members and policy-makers will add important nuance in future research.

## Conclusion

 The early developmental stage of Australian AHSCs presents an important and timely opportunity for formative learning and evaluation to optimise knowledge mobilisation processes towards achieving meaningful impact. This requires a focus on action-oriented roles and processes needed to deliver strategic goals and on organisational mechanisms to deliver health systems impact within diverse local contexts.

## Acknowledgements

 The authors thank the interviewees from the participating AHSCs who generously offered their time for this research.

## Ethical issues

 Ethics approval for the study was obtained from the University of Adelaide Human Research Ethics Committee (H-2018-239).

## Competing interests

 Authors declare that they have no competing interests.

## Authors’ contributions

 AE contributed to study design and data analysis and produced the first draft of the manuscript. GH, RCW, and AE undertook data collection in the study. RCW, MF, RK, AK, IM, and HS contributed to study design and data analysis and provided critical feedback on manuscript drafts. GH oversaw all aspects of the study including study design, data collection and analysis and contributed to drafting the manuscript.

## Funding

 This study was supported by a University of Adelaide Faculty of Health and Medical Sciences Establishment Grant. The grant supported travel of the project team to attend in-person meetings and workshops relating to the study.

 RK is part-funded by the National Institute for Health Research Applied Research Collaboration (NIHR ARC) Greater Manchester. The views expressed in this article are those of the authors and not necessarily those of the National Health Service, the NIHR, or the Department of Health and Social Care.

## Authors’ affiliations

^1 ^College of Public Health, Medical and Veterinary Sciences, James Cook University, Townsville, QLD, Australia. ^2^Australian Institute of Health Innovation, Macquarie University, Sydney, NSW, Australia. ^3^Centre for Sustainable Human Resource Management and Wellbeing, Australian Catholic University, Melbourne, VIC, Australia. ^4^Manchester Metropolitan University, Manchester, UK. ^5^The University of Manchester, Manchester, UK. ^6^Caring Futures Institute, College of Nursing and Health Sciences, Flinders University, Adelaide, SA, Australia. ^7^Monash Business School, Monash University, Melbourne, VIC, Australia. ^8^Monash Centre for Health Research and Implementation, Monash University, Melbourne, VIC, Australia. ^9^Warwick Business School, University of Warwick, Coventry, UK. ^10^Adelaide Nursing School, University of Adelaide, Adelaide, SA, Australia. ^11^Australian Centre for Health Services Innovation, Queensland University of Technology, Brisbane, QLD, Australia.

## References

[1] Theile DE, Scott IA, Martin JH, Gavrilidis A (2014). Enabling the success of academic health science centres in Australia: where is the leadership?. Med J Aust.

[2] Fisk NM, Wesselingh SL, Beilby JJ (2011). Academic health science centres in Australia: let’s get competitive. Med J Aust.

[3] Dickinson H, Ledger J (2018). Accelerating research translation in healthcare: the Australian approach.

[4] Kislov R, Wilson PM, Knowles S, Boaden R (2018). Learning from the emergence of NIHR Collaborations for Leadership in Applied Health Research and Care (CLAHRCs): a systematic review of evaluations. Implement Sci.

[5] French CE, Ferlie E, Fulop NJ (2014). The international spread of Academic Health Science Centres: a scoping review and the case of policy transfer to England. Health Policy.

[6] Edelman A, Taylor J, Ovseiko PV, Topp SM (2018). The role of academic health centres in improving health equity: a systematic review. J Health Organ Manag.

[7] Ferlie E, Nicolini D, Ledger J, D’Andreta D, Kravcenko D, de Pury J (2017). NHS top managers, knowledge exchange and leadership: the early development of Academic Health Science Networks–a mixed-methods study. Health Serv Deliv Res.

[8] Rycroft-Malone J, Burton CR, Wilkinson J (2016). Collective action for implementation: a realist evaluation of organisational collaboration in healthcare. Implement Sci.

[9] Fitzgerald L, Harvey G (2015). Translational networks in healthcare? evidence on the design and initiation of organizational networks for knowledge mobilization. Soc Sci Med.

[10] Bradshaw C, Atkinson S, Doody O (2017). Employing a qualitative description approach in health care research. Glob Qual Nurs Res.

[11] Bornbaum CC, Kornas K, Peirson L, Rosella LC (2015). Exploring the function and effectiveness of knowledge brokers as facilitators of knowledge translation in health-related settings: a systematic review and thematic analysis. Implement Sci.

[12] Oborn E, Barrett M, Prince K, Racko G (2013). Balancing exploration and exploitation in transferring research into practice: a comparison of five knowledge translation entity archetypes. Implement Sci.

[13] Gulati R, Wohlgezogen F, Zhelyazkov P (2012). The two facets of collaboration: cooperation and coordination in strategic alliances. Acad Manag Ann.

[14] Lockett A, El Enany N, Currie G (2014). A formative evaluation of Collaboration for Leadership in Applied Health Research and Care (CLAHRC): institutional entrepreneurship for service innovation. Health Serv Deliv Res.

